# Analytical validation of the Target Selector ctDNA platform featuring single copy detection sensitivity for clinically actionable *EGFR*, *BRAF*, and *KRAS* mutations

**DOI:** 10.1371/journal.pone.0223112

**Published:** 2019-10-03

**Authors:** Jason C. Poole, Shan-Fu Wu, Timothy T. Lu, Cecile Rose T. Vibat, Anh Pham, Errin Samuelsz, Manisha Patel, Jeffrey Chen, Tony Daher, Veena M. Singh, Lyle J. Arnold

**Affiliations:** 1 Biocept, Inc., San Diego, California, United States of America; 2 Aegea Biotechnologies, Inc., San Diego, California, United States of America; The Ohio State University, UNITED STATES

## Abstract

**Background:**

Personalized medicine requires accurate molecular profiling for targeted therapy decisions. Insufficient tissue yield or tumor heterogeneity frequently limits the correct tissue biomarker determination. As a noninvasive complement to traditional tissue biopsies, liquid biopsies detect and track cancer driver mutations from biofluids (e.g., blood, urine). Here we present the analytical validation of Target Selector^™^ ctDNA assays capable of single mutant DNA copy detection.

**Methods:**

The Target Selector ctDNA assay applies a patented Switch-Blocker technology to suppress amplification of background (wild-type) WT alleles, while allowing specific amplification of very low frequency mutant alleles. In contrast to allele specific enrichment technologies like ddPCR, one Switch-Blocker inhibits amplification of a DNA target up to 15 bp in length (e.g., one Switch-Blocker covers all *KRAS* exon 2, codon 12 and 13 variants). Target enrichment is achieved through a quantitative PCR reaction; subsequent DNA sequencing confirms mutation identity. Analytical validation with cancer cell line DNA was conducted by three independent operators using five instruments across five days.

**Results:**

A total of 3086 samples were tested on *EGFR*, *BRAF* and *KRAS* Target Selector ctDNA assays, with *EGFR* WT as a reference. All assays showed >99% analytical sensitivity and specificity. Single mutant copy detection is confirmed by experimental data and theoretical estimates. In the presence of 14000 WT DNA copies, limits of detection were: *EGFR* Del19, 0.01%; *EGFR* L858R, 0.02%; *EGFR* T790M, 0.01%; *BRAF* V600E, 0.01%; *KRAS* G12C, 0.02%. Inter- and intra-assay analyses showed r^2^>0.94, suggesting consistent performance among operational variables. Healthy donor samples (100 tests) showed clinical specificity at >99%. Finally, Target Selector clinical experience data of >2200 patient samples is consistent with published tissue mutation prevalence.

**Conclusions:**

Highly sensitive Target Selector ctDNA assays with single mutant copy detection and limit of detection at 0.02% or better enable accurate molecular profiling vital for disease management.

## Introduction

Tumor tissue has traditionally been required for both cancer diagnosis and molecular biomarker testing. All too often, acquired tissue is exhausted during the initial diagnosis, leaving insufficient tissue for subsequent biomarker testing. This poses a challenge for healthcare professionals making therapeutic decisions since the patient has a confirmed disease, yet biomarker status is lacking to guide the best course of treatment. Another pitfall of tissue biopsy is the possibility of missing intra- and inter- tumor heterogeneity, where a spectrum of molecular alterations may impact a patient’s cancer. This heterogeneity presents clinical challenges in determining the best treatment options for patients, as well as evaluating clonal evolution related to acquired resistance and progression [1, 2].

The use of liquid biopsies for precision medicine (the stratification of patients using biomarkers associated with targeted therapies) is an emerging trend in oncology that is gaining adoption for guiding therapeutic decisions in cancer management. Both domestic and international clinical practice guidelines have recently been updated to support the testing of plasma derived circulating tumor DNA (ctDNA) [3, 4]. Testing is also recommended in some circumstances to evaluate molecular markers related to targeted therapies with regulatory approval for lung cancer [3, 4]. Traditionally, the practice of clinical oncology utilizes tumor tissue biopsy as the gold standard for molecular profiling. Liquid biopsy can play an important role in determining the best targeted therapy when tissue is insufficient for both diagnosis and biomarker testing. Lung cancer is the most common example of this issue due to the limitations posed by the location of the tumor(s) and/or the small quantity of tissue that can be obtained via fine needle aspiration. While a second, more invasive biopsy may be justified to acquire additional tissue for molecular testing, these procedures are costly and can subject the patient to significant risk [5, 6]. In such circumstances, as well as when a patient is unwilling or too ill to undergo surgical procedures, liquid biopsy is a viable means to provide additional actionable information. Similarly, tissue re-biopsy may provide essential biomarker information on new lesions when patients relapse, but the physician must weigh the risk of biopsy and its associated complications versus the need to obtain additional biomarker information with the potential to change the course of treatment [7]. When used as a complement to tissue analyses or as a monitoring tool, liquid biopsy offers a non-invasive and systemic approach to identify tumor mutations by assessing ctDNA released from tumor cells into circulation. Liquid biopsies and enrichment strategies like Target Selector are also applied in fields outside of oncology, being utilized in routine clinical practice for infectious disease, prenatal diagnosis, and transplantation biology [8–11].

Precision medicine requires the accurate detection of actionable mutations in patients with cancer. In conjunction with providing information for personalized targeted therapies [12], serial liquid biopsy testing has the potential to monitor therapeutic response, minimal residual disease, and disease recurrence [13–15]. These factors impact real world clinical applications and healthcare economics, often being the fastest means towards biomarker resolution, as well as the most economical and least invasive testing option. For these reasons, the clinical application of liquid biopsy for cancer management is increasingly supported in the literature [15–20].

**Fig 1:**
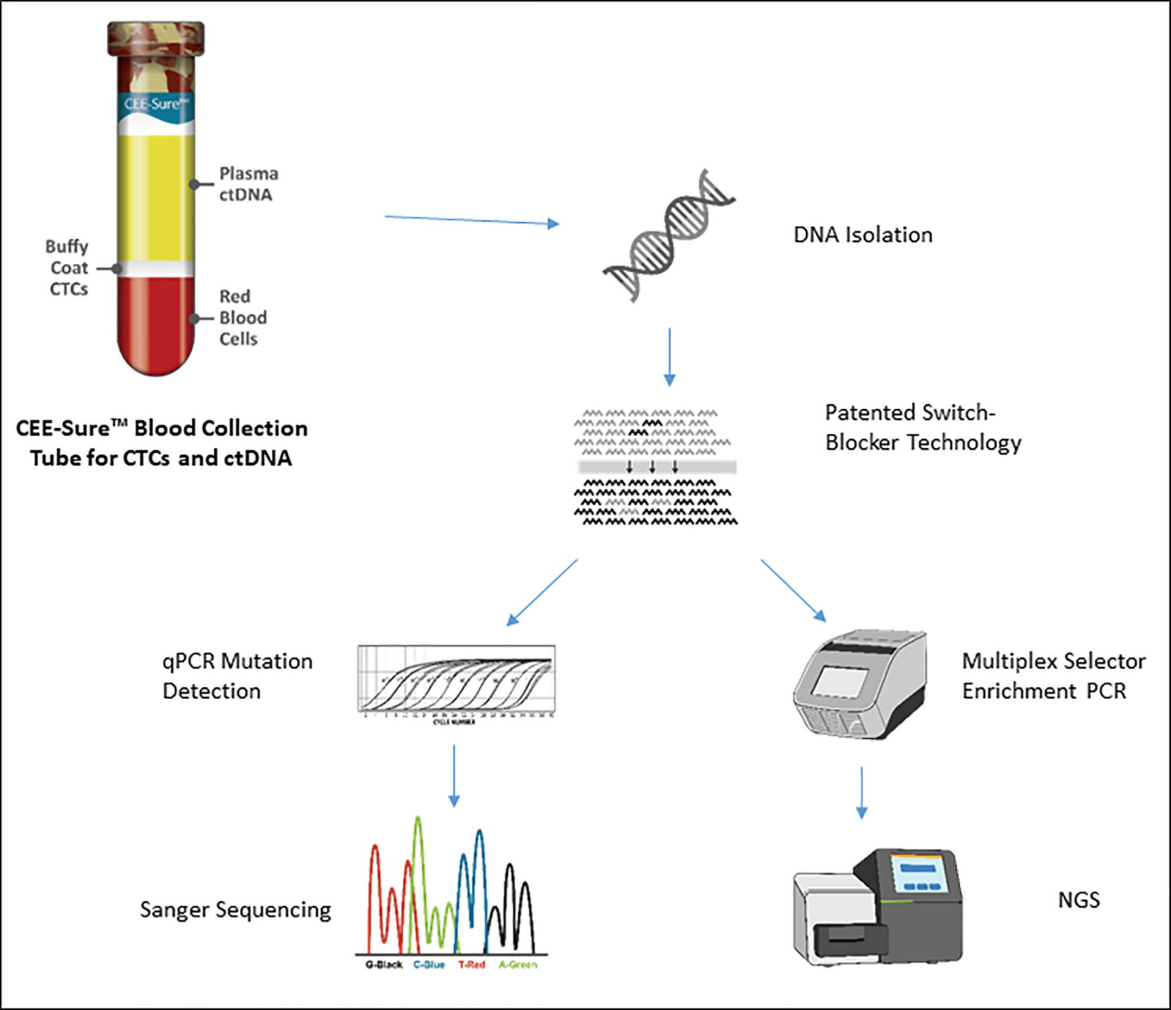
Target Selector workflow. Selective mutation enrichment enables highly sensitive ctDNA analysis by utilizing a qPCR enrichment and quantitation step followed by Sanger or NGS DNA sequencing to verify mutations. Performing PCR upfront and then sequencing delivers added confidence to final results. Switch-Blocker technology enriches oncogene mutations and suppresses WT DNA resulting in ultra-high sensitivity and specificity. All ctDNA tests are quantitative and can be used to monitor mutation load. NGS technology allows multiplexing capabilities and future panel development.

Because genomic alterations from solid tumors are present in minute amounts in peripheral blood, an ultra-sensitive limit of detection (LoD) is essential for precise liquid biopsy testing at low mutant allele fractions (MAFs) in circulation. Detecting ultra-low levels of gene mutations in ctDNA is achieved with the Target Selector ctDNA assay using real-time PCR based assays in conjunction with a patented “Switch-Blocker” methodology to suppress WT DNA, while allowing specific amplification of mutations in the target region [21]. The Target Selector ctDNA assay utilizes a blocking probe strategy of Switch-Blockers to enrich for rare mutations and simultaneously quantify mutational load through qPCR. Additionally, sequencing is performed for orthogonal confirmation of the test result (Fig 1).

We focused on five important and clinically actionable gene targets (*EGFR* (Exon 19 deletions, L858, and T790), *BRAF* (V600), and *KRAS* (G12/G13)), whose alterations are particularly relevant to lung cancer, melanoma, and colorectal cancer.

## Results

### Target Selector ctDNA assay overview

**Fig 2:**
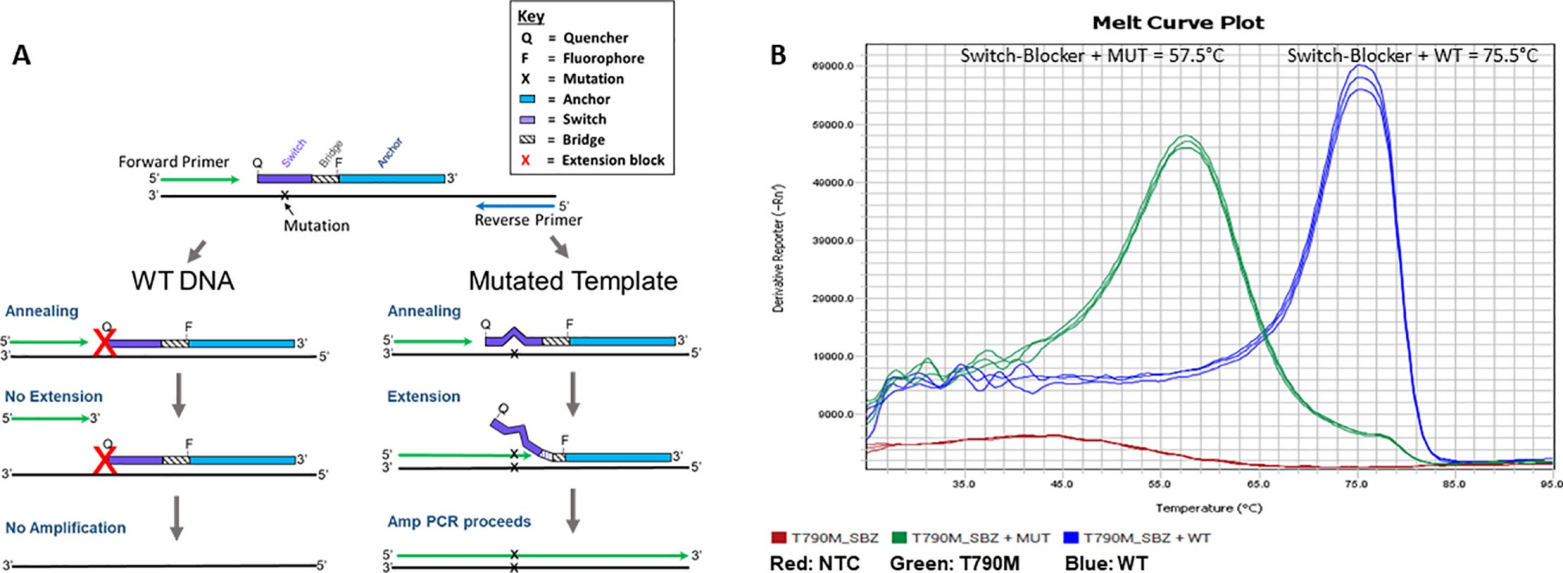
Target Selector mechanism and melting temperature analysis. (A) The Target Selector ctDNA assays utilize qPCR and a patented Switch-Blocker molecule for mutation enrichment. The assays are targeted mutation tests which apply a blocker (Switch + Anchor) to block WT DNA amplification while allowing mutant DNA amplification. One specific blocker covers variants on a short stretch of target DNA (up to 15 bp for nucleotide variants). The legend summarizes key features of this design. (B) Melt curve analysis of the EGFR T790M Switch region showing a melt peak of 57.5 C° when bound to a mutant template containing a single nucleotide mismatch (green) but a 75.5 C° melt peak when hybridized to the fully complementary WT DNA (blue). The “No Template Control” (NTC) (red) is quenched across all temperatures.

When the Target Selector Switch-Blocker hybridizes to amplification products at lower temperatures (50–55°C range), the quencher and fluorophore are separated, leading to a fluorescent signal. When the Switch opens, fluorescence is quenched significantly as the quencher comes in close vicinity to the fluorophore. When the entire Switch-Blocker fully separates from the target, additional quenching of the fluorophore occurs (Fig 2A). When the anchor portion of the Switch-Blocker hybridizes, the local concentration of the Switch region, near its target, is increased about 10,000 fold. This increase in Switch concentration raises its Tm to a perfectly matched target (wild-type) by 30°C or more. When combined with other Switch modifications to increase affinity, the T_m_ of the 7–15 nucleotide Switch region is increased to an even greater degree (~75°C). In total, these modifications create up to a 20°C difference in hybridization T_m_ of the switch when bound to WT as compared to when a mutation is present. (See Fig 2B).

**Fig 3:**
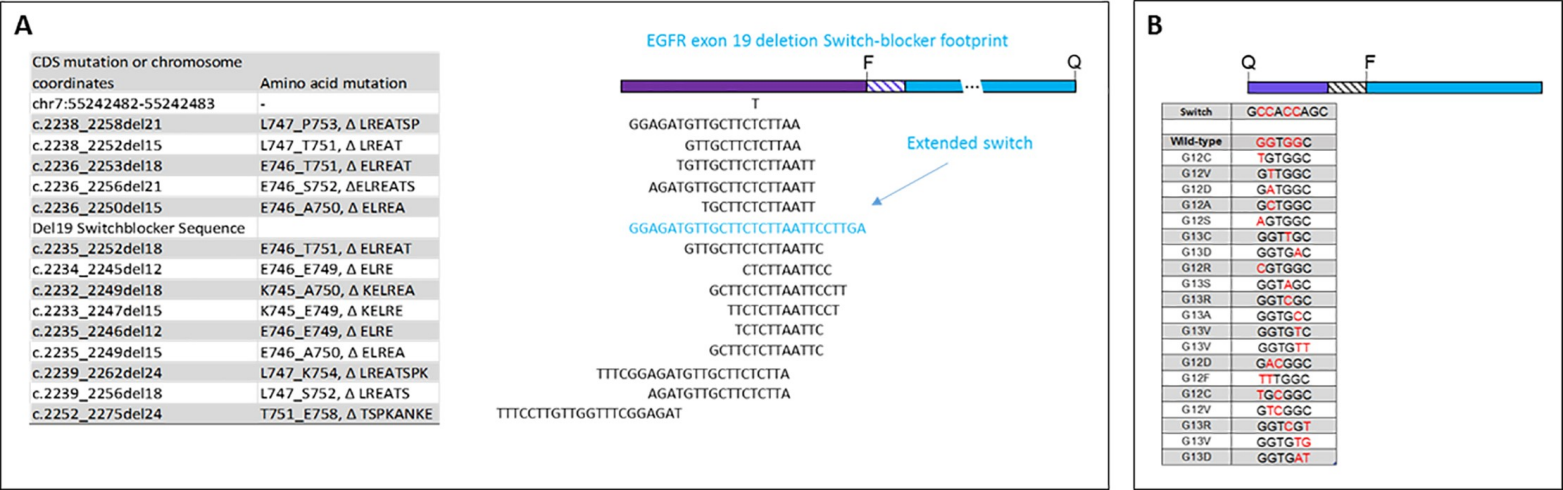
Enrichment region of Del19 and KRAS Switch-Blockers. (A) Small insertions and deletions can be detected with a single Switch, extended to account for a larger footprint of the alterations if needed. Switch-Blockers are built to detect single point mutations but are even more efficient when detecting alterations containing more than one nucleotide. (B) The Target Selector ctDNA assays apply a specific blocker to cover variants on a short stretch of target DNA (up to 15 bp for nucleotide variants). For example, one KRAS exon 2 blocker covers all variants on both G12 and G13 codon positions.

The typical length of the Switch region is between 7–15 nucleotides with a versatile design, whereby lengthening or shortening of the Switch depends on the context of the alteration. Length of the Anchor and modified nucleotides can be implemented within the Switch-Blocker sequence to make T_m_ adjustments as needed for accommodating Switch length, assay temperatures for optimal enrichment, and the position of the Switch. Fig 3 shows two examples of how the technology can be applied. Fig 3A shows the *EGFR* exon 19 deletion Switch-Blocker footprint. This Switch-Blocker is an example with an extended Switch. Because the technology is optimized and sensitive to even a single nucleotide change, detection of larger regions such as an insertion or deletion becomes trivial. Furthermore, to enable quantification by real-time PCR, reporting elements of the EGFR exon 19 deletion Switch-Blocker were moved entirely to the Anchor portion, with fluorescent reporting being controlled by an internal hairpin called a “flip” probe. Fig 3B shows various mutants in the *KRAS* exon 2 region which has a standard Switch length, with the quencher and fluorophore spanning the Switch and Bridge regions. In all, up to 40 different mutations have been sequenced in this region, all within a 5-nucleotide span in codons 12 and 13 of exon 2. All of these SNVs are enriched by a single *KRAS* Switch-Blocker.

### Testing the impact of WT DNA

**Fig 4:**
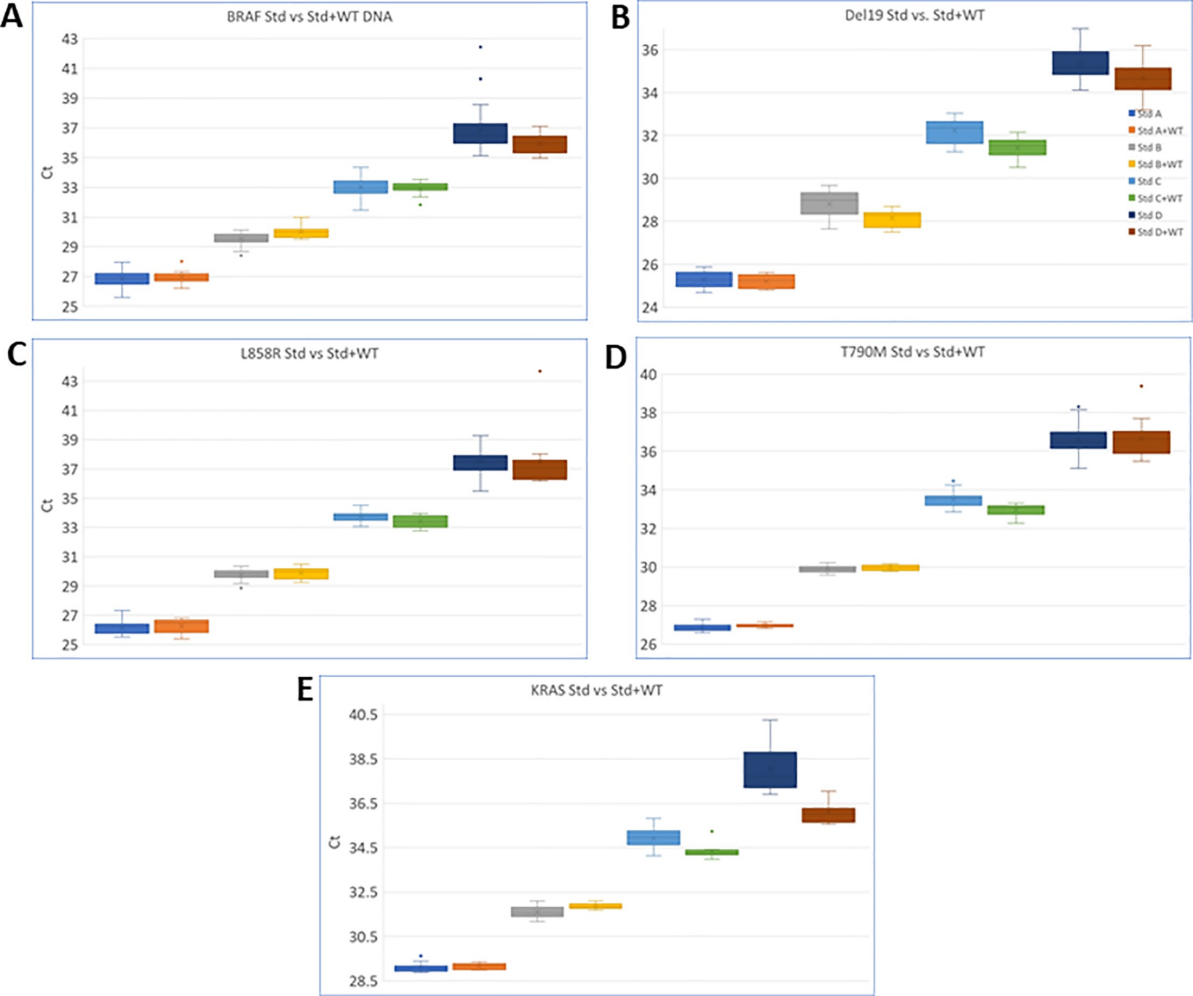
Box and whisker plots of each of the 5 assays performed in the presence or absence of 14,000 copies of WT DNA. (A)BRAF (B) EGFR exon 19 deletion (C) EGFR L858R (D) EGFR T790M (E) KRAS exon 2. Stds A,B,C, and D are presented with and without WT DNA as shown in the legend. The boxes represent the interquartile range for the 25th-75th percentile and the edge of the whiskers show minimum and maximum values excluding outliers. The middle box line is the median and the X inside the boxes represents the mean while any outliers are printed as colored dots outside the range.

The Target Selector test is designed to suppress WT and amplify only mutant DNA molecules. To test this, we modeled WT DNA as an interfering substance. We built a series of ddPCR verified standards which ranged in concentration from Standard A at approximately 5000 mutant copies per reaction down to Standard G which contained only a single mutant copy (Table 1). We then tested these standards with or without an added 50ng (approximately 14,000 copies) of WT DNA to determine its impact on signal intensity during qPCR enrichment. As indicated by the emergent threshold cycle for each condition (Table 2), none of the assays showed a significant impact on performance or degradation resulting from the additional WT DNA. In most cases, at the extreme low end of mutant copy input, overall Ct strength improved slightly to moderately with the addition of WT DNA. This indicates rare mutations may amplify more efficiently when WT DNA is present (see Fig 4, Std D and Std D + WT for examples). The cause of this is uncertain, but we hypothesize having an excess complement of newly synthesized reverse strands leads to somewhat earlier Ct values. Regardless, all samples of pure mutant input amplified close to their counterparts with the large background of WT DNA, demonstrating an average delta Ct 0.32 Cts and a standard deviation of +/- 0.52 Cts across all concentrations measured. This demonstrates the ability of the Target Selector technology to effectively block WT and focus exclusively on the enrichment and amplficiation of mutations of interest. This effect provides the foundation for detecting vanishingly low mutant copy inputs irrespective of WT DNA concentrations found in the sample.

**Table 1:** Mutant reference standard input was established using ddPCR. Replicates are increased at the lowest concentrations to account for stochastic effects.

Name	Mutant Input	WT input (human placental DNA)	Replicates per Standard
Standard A	~5120 copies	~14,000 copies	3
Standard B	~640 copies	~14,000 copies	3
Standard C	~80 copies	~14,000 copies	3
Standard D	~10 copies	~14,000 copies	3
Standard E	1:2 of Std D	~14,000 copies	20
Standard F	1:2 of Std E	~14,000 copies	20
Standard G	1:2 of Std F	~14,000 copies	20

**Table 2:** Target Selector performance at low copy number with and without WT DNA background. Through comparison of the ‘Mean of Standard’ and ‘Mean of Standard with WT’, we obtain the delta Cts. Standards with and without a WT DNA background are largely equivalent, with an average delta Ct of 0.43 (as absolute values) and Standard Deviation of 0.52 Cts.

Target	Sample Name	Mean of Standard Ct	Mean of Standard plus WT	Delta Ct
BRAF	Std A	26.97	26.97	0
BRAF	Std B	29.61	29.99	-0.38
BRAF	Std C	33.09	32.91	0.18
BRAF	Std D	36.6	35.94	0.66
BRAF	Std F	38.19	38.06	0.14
Del19	Std A	25.29	25.21	0.07
Del19	Std B	28.82	28.15	0.67
Del19	Std C	32.23	31.43	0.79
Del19	Std D	35.34	34.68	0.65
Del19	Std F	36.85	36.23	0.62
L858R	Std A	26.15	26.26	-0.11
L858R	Std B	29.76	29.9	-0.14
L858R	Std C	33.72	33.44	0.29
L858R	Std D	37.5	36.91	0.59
L858R	Std F	39.39	38.95	0.44
T790M	Std A	26.9	26.95	-0.06
T790M	Std B	29.94	29.97	-0.02
T790M	Std C	33.56	32.96	0.6
T790M	Std D	36.67	36.65	0.01
T790M	Std G	38.69	39.05	-0.36
KRAS	Std A	29.08	29.17	-0.09
KRAS	Std B	31.59	31.86	-0.26
KRAS	Std C	34.92	34.32	0.60
KRAS	Std D	38.04	36.10	1.94
KRAS	Std F	39.26	38.14	1.12

### Single copy sensitivity and Poisson distribution with maximum likelihood estimates

In a liquid biopsy, tumor tissue derived mutations circulate in a vast excess of WT DNA released by healthy tissue from locations throughout the body. Accordingly, the most critical feature of a liquid biopsy test is its sensitivity, which determines its effectiveness to detect rare somatic variants. Here we demonstrate that our Target Selector assays have single copy sensitivity, the lowest limit of detection possible for any DNA-based diagnostic test. To evaluate detection over a wide dynamic range down to a single copy, we used cell line DNA or synthetic gBlock controls (IDT DNA, Inc., San Diego, California, USA) containing a variety of mutations falling within the Switch region undergoing validation. Selecting common mutant variants for further study, droplet digital PCR (ddPCR) was used to quantify reference DNA standards (Table 3, ‘ddPCR Copies (Reference)’), serving as an orthogonal means to confirm copy numbers determined by Target Selector. Table 3 shows experimental results where Target Selector was used to quantify a 2-fold dilution series of standard D (Std D) containing decreasing copy numbers of mutations, with or without a large background of WT DNA. Standards E, F, and G are 2-fold serial dilutions of Std D expected to contain approximately 4, 2, and 1 copies of mutation per reaction respectively. Based on Poisson statistics, only a fraction of test wells will contain a DNA fragment at these low copy levels. [22, 23]. This is consistent with experimental observations (Table 3, Columns ‘Empty Wells/Total Replicates’ and ‘Actual Percent Empty Wells’).

**Table 3:** Determining sensitivity of the Target Selector Assay at or near single copy input levels. Column ‘ddPCR Copies (Reference)’ shows ddPCR validation of controls for cell lines containing mutations of interest. Source DNA was isolated from named cell lines or commercially sourced human placental DNA. Copy numbers per reaction for Standards A-D were determined by independent measurements post dilution using droplet digital PCR. Target Selector results at stochastic sampling levels show qPCR copies as determined by the maximum likelihood estimate of the standard curve, the number of empty well and replicates run per concentration, the percentage of empty wells and the copy number as calculated using the Poisson statistic.

	Sample Name	ddPCR Copies (Reference)	Target Selector qPCR Copies (MLE)	Empty Wells/ Total Replicates	Actual Percent Empty Wells	Calculated Poisson Copies
***BRAF Source*: *SK-MEL-28***	Std A	4510	** **	** **	** **	** **
	Std B	515	** **	** **	** **	** **
	Std C	64	68.55	0/18	0.0%	>10
	Std D	7	7.37	0/18	0.0%	>10
	Std E	(3.5)	2.93	5/36	13.9%	1.97
	Std F	(1.75)	2.57	18/60	30.0%	1.2
	Std G	(0.875)	1.59	26/60	43.3%	0.84
**EGFR Del19 Source: H1650**	Std A	5337	** **	** **	** **	** **
	Std B	571	** **	** **	** **	** **
	Std C	78	62.3	0/18	0.0%	>10
	Std D	(6)	8	0/18	0.0%	>10
	Std E	(3)	4.64	0/36	0.0%	>10
	Std F	(1.5)	3.5	10/60	16.7%	1.79
	Std G	(0.75)	2.37	25/60	41.7%	0.88
***EGFR L858R Source*: *H1975***	Std A	5125	** **	** **	** **	** **
	Std B	609	** **	** **	** **	** **
	Std C	77	80.7	0/18	0.0%	>10
	Std D	11	11.72	0/18	0.0%	>10
	Std E	(5.5)	6.47	2/36	5.6%	2.89
	Std F	(2.75)	4.71	10/60	16.7%	1.79
	Std G	(1.375)	3.17	21/60	35.0%	1.05
***EGFR T790M Source*: *H1975***	Std A	5164	** **	** **	** **	** **
	Std B	609	** **	** **	** **	** **
	Std C	91	74.93	0/18	0.0%	>10
	Std D	9	11.03	0/18	0.0%	>10
	Std E	(4.5)	5.81	3/36	8.3%	2.48
	Std F	(2.25)	3.69	7/60	11.7%	2.15
	Std G	(1.125)	3.03	27/60	45.0%	0.8
***EGFR WT Source*: *Human Placental DNA***	Std A	13980	** **	** **	** **	** **
	Std B	1536	** **	** **	** **	** **
	Std C	200	130.35	0/18	0.0%	>10
	Std D	19	26.53	0/18	0.0%	>10
	Std E	(9.5)	13.5	0/24	0.0%	>10
	Std F	(4.75)	7.91	5/40	12.5%	2.08
	Std G	(2.375)	4.15	14/40	35.0%	1.05
***KRAS exon 2 Source*: *H2122***	Std A	5048	** **	** **	** **	** **
	Std B	585	** **	** **	** **	** **
	Std C	82	70.3	0/18	0.0%	>10
	Std D	10	12.21	0/18	0.0%	>10
	Std E	(5)	6.93	5/36	13.9%	1.97
	Std F	(2.5)	4.57	18/60	30.0%	1.2
	Std G	(1.75)	4.37	26/60	43.3%	0.84

Table 3 also reports the input quantities of each test as interpolated from the observed qPCR data using a maximum likelihood estimator (MLE) and the fractional dropout rate. The estimator uses the negative log likelihood of the observed distribution matching the Poisson distribution. Calculating the estimated Poisson copy numbers using this method is more accurate than using the percent of replicates with zero copies since it makes use of multiple estimates observed at each standard level. The empirical values and the magnitude relationships of estimated copy numbers between standard levels provide strong evidence of the capability to detect mutants in an extremely low target copy range. Here we show this relationship is effective down to a single copy as demonstrated by the similarity between extrapolated copies for the ddPCR reference and calculated Poisson copies.

Table 4 lists the theoretical percentage of empty wells expected when predicting specific copy numbers in the specified reaction volume. For example, 36.79% of wells would be empty, given the input of a single copy per reaction across many reactions. If the number of experimental positives matched the expected value of positives in the corresponding Poisson distribution, then the assay was considered proficient for detection at that concentration in analytical validation testing.

**Table 4:** Theoretical expectation of empty wells by copy number input according to a Poisson distribution. For example, when pipetting an average of 1 copy per reaction into 100 wells, the expected number of empty wells would be 37.

Average copy input per well	Theoretical percent of replicates with 0 copies
1	36.79%
2	13.53%
3	4.98%
4	1.83%
5	0.67%
6	0.25%
7	0.09%
8	0.03%
9	0.01%
10	0.00%

**Fig 5:**
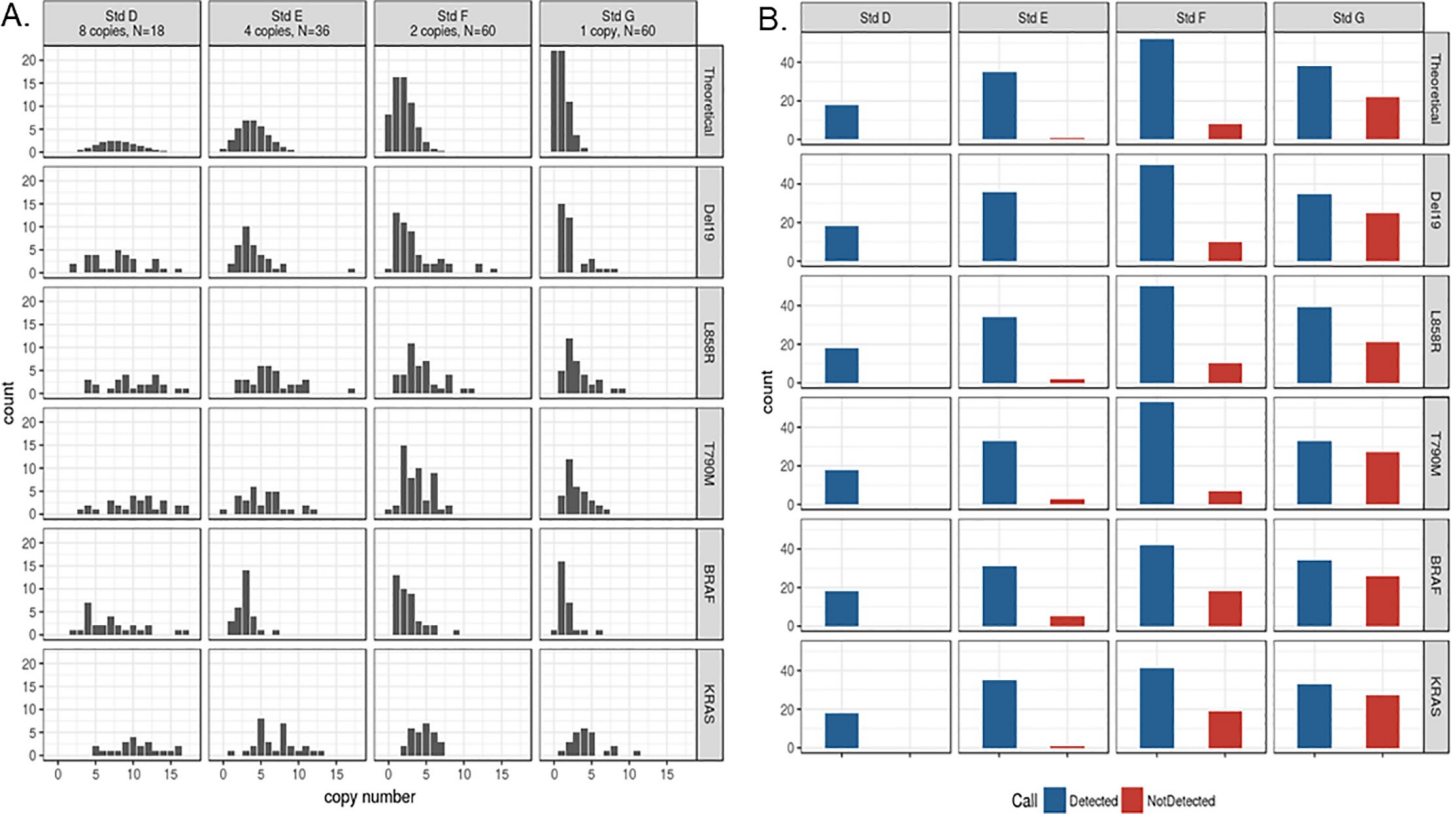
Poisson and single copy detection. Detection sensitivity of each ctDNA assay was determined through a serial dilution of mutant copies (in the absence of WT copies). A. Histograms for the frequency of copy number detection occurrences. B. Plots for the number of detection occurrences. The top rows show the theoretical profiles based on a Poisson distribution, with corresponding sample numbers (N) for each standard. The actual data of each assay matches well with the theoretical model.

Fig 5A shows graphical representations of Poisson distributions for Standards D, E, F, and G compared to Target Selector detection results of this dilution series. For each mutant assay, the experimental data recapitulate well with the theoretical calculations; the experimental data show a distribution aligning with the theoretical model at each mutant input level. Detection vs. dropout rates are shown Fig 5B. As expected, as the dilution increases and copy number decreases, anticipated experimental drop out events parallel the theoretical Poisson distribution (Fig 5B, red bars).

### Analytical sensitivity and specificity

We compared the performance of our Target Selector assay against ddPCR orthogonally validated controls to measure analytical sensitivity and specificity. When sheared cancer cell-line DNA was spiked into a background of 14,000 copies of sheared human placental DNA, each of our tests demonstrated an analytical sensitivity of 0.02% or better (Table 5). One *BRAF* false negative was observed out of 135 positive samples tested. The overall false negative rate was 1 out of 667 tests.

**Table 5:** Analytical sensitivity, specificity and LOD for all 5 Target Selector ctDNA Assays. Each Biocept Target Selector ctDNA assay was analytically validated and showed >99% analytical sensitivity and >99% analytical specificity. Limit of detection (LOD) for each assay was tested in the presence of 14,000 WT copies and showed sensitivity at 0.02% MAF or better. FN, false negative; TP, true positive; FP, false positive; TN, true negative.

ctDNA Assay	Analytical Sensitivity (FN/TP)	Analytical Specificity (FP/TN)	LOD
***EGFR* Del19**	> 99% (0/120)	> 99% (1/112)	0.01%
***EGFR* L858**	> 99% (0/138)	> 99% (1/112)	0.02%
***EGFR* T790**	> 99% (0/138)	> 99% (0/112)	0.01%
***BRAF* V600**	> 99% (1/135)	> 99% (0/112)	0.01%
***KRAS* exon 2**	> 99% (0/136)	> 99% (0/112)	0.02%

Analytical specificity was determined by testing 100 WT samples each containing 50ng of human placental DNA across each of the assays. The number of true negatives and false positives were calculated to determine the specificity of the assay. A true negative was defined as trending below the established fluorescent threshold for detection in qPCR. If no Ct emerged, then the sample was considered negative and no sequencing was required. In a few cases (Table 6), qPCR Cts emerged, but evaluation of their melt curves revealed non-specific products, and thus were excluded. In a few other samples, the product fell within the melt curve criteria and they were subjected to Sanger Sequencing. In cases where samples sequence as WT, they are considered true negatives. This orthogonal confirmation of Ct, Melt Curve and Sequencing is valuable in tests like *EGFR*, *KRAS* and *BRAF* since they are part of large gene families that have many pseudogenes. Having these additional criteria provides a cross check to what might otherwise become false positive signals in the highly diverse and genetically unstable genomes of cancer patients. They are especially valuable in the context of an enrichment platform like Target Selector Since any mutation under the switch will be enriched regardless of its relevance to cancer.

**Table 6:** Number of qPCR traces that amplify for all WT samples prior to melt curve analysis or sequencing. Eg. qPCR specificity prior to sequencing. qPCR provides quantitative copy number data and positives are orthogonally confirmed using sanger sequencing. This helps to filter non-specific qPCR signals and/or confirms which nucleotide is mutant.

	Validation Plate Number					
Target Name	1	2	7	8	9	10
BRAF	2/50	0/50	0/3	0/3	1/3	0/3
Del19	3/50	1/50	0/3	1/3	1/3	0/3
L858R	3/50	3/50	0/3	0/3	0/3	1/3
T790M	5/50	4/50	2/3	0/3	1/3	0/3
KRAS	0/50	0/50	0/3	0/3	0/3	0/3

After all criteria were considered, specificity for this test group was >99% for all targets. Only two false positives were detected in 560 tests across all five assays after the full protocol of qPCR, Sanger Sequencing and melting temperature cutoffs were applied. To determine clinical specificity, plasma samples from 20 healthy donors were processed; cell-free DNA (cfDNA) was isolated from 4mL of plasma using a cfDNA extraction protocol (QIAGEN, Germantown, Maryland, USA) on a QIAsymphony instrument (QIAGEN). Samples were subsequently evaluated using each of the 5 Target Selector mutation tests in separate reactions to look for false positive signals. *EGFR* WT was run using a Switch-Blocker with a built-in mismatch to WT DNA. This was used to quantitate the DNA yield extracted from each individual, calculate the total genomic equivalents and confirm that DNA was present. No mutations were detected in these samples and sequence positives were not observed for any of the assays run on healthy donor specimens. Therefore, clinical specificity was >99% for all Target Selector assays. Results are summarized in Table 7 below.

**Table 7:** Clinical specificity for each Target Selector ctDNA assay. Clinical specificity of each Target Selector ctDNA assay was validated on DNA extracted from 4ml of healthy donor plasma samples. For each of Target Selector ctDNA assay, no mutation was detected from 20 healthy donors, suggesting >99% clinical specificity.

ctDNA Assay	Tested	Detected	Clinical Specificity
***EGFR* Del19**	20	0	> 99%
***EGFR* L858**	20	0	> 99%
***EGFR* T790**	20	0	> 99%
***BRAF* V600**	20	0	> 99%
***KRAS* exon 2**	20	0	> 99%

### Inter-assay and intra-assay precision (reproducibility)

To assess the reproducibility of our assays, a 4-point standard curve was built from either genomic cell line DNA containing known target mutations, or commercially obtained human placental DNA considered as WT. The cell line SK- MEL-28 was used for *BRAF*, H1975 for *EGFR* T790M and L858R, H1650 for *EGFR* exon 19 deletion, H2122 for *KRAS* exon 2 and human placenta genomic DNA. The standard curve was run at 8-fold dilutions in triplicate across 5 instruments by 2 different users (4x3x5x2 = 120 samples total). All standard curve samples were prepared and qualified using ddPCR to verify copy number inputs of the control materials. In addition, triplicate NTC reactions and 50ng human placenta control samples were run. The experiment was completed on non-consecutive days by two different laboratory technologists. To pass reproducibility criteria, every run had to fall within 1 Ct standard deviation of each other along each point of the curve. Inter-assay reproducibility was measured between plates, instruments and operators with similar results though there was slightly higher variability, (not statistically significant), when grouped by operator (S1 Table). Similarly, we analyzed the intra-assay reproducibility using the same data set, where replicates within each run were analyzed. Each standard was run at least 5 times in triplicate at each standard concentration and the intra-assay reproducibility for these tests fell within a standard deviation of <1 Ct for 118 out of 120 trials (S2 Table).

### Reportable range

**Fig 6:**
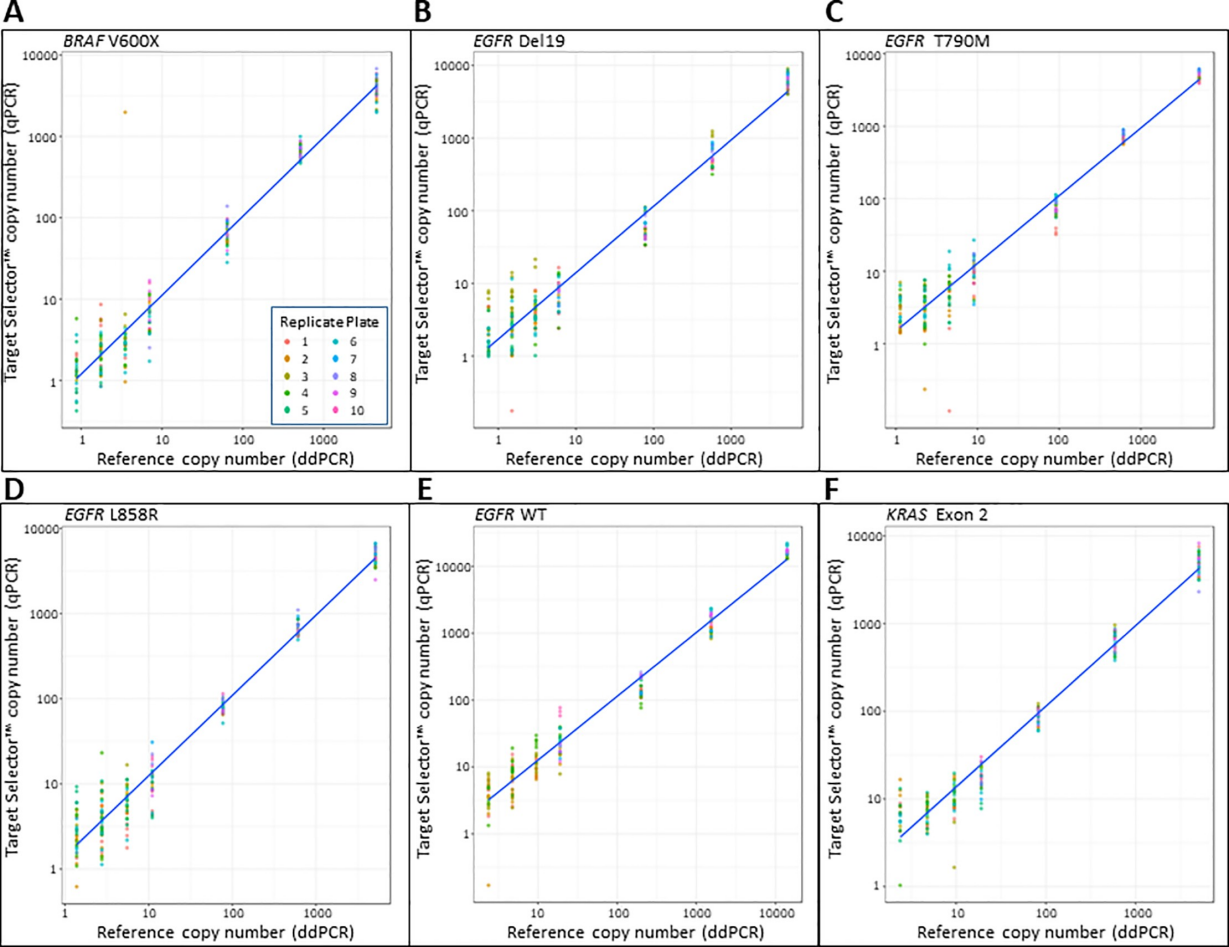
Linearity and regression plots. These plots show the regression curves derived from reference standards across 10 plates after melting temperature and minimum Ct (>10) quality measures were applied. Panels A-F show the standard curve data for each target plotted onto a single graph from standards A-G. Inputs for each target range from over 5000 copies on the far right down to or near a single copy on the far left. Reference copy numbers along the x-axis are derived from measured data using ddPCR for each of the 4 most concentrated standards (A-D) or calculated based on dilution from standard D for standards E, F and G. Y-axis values are derived from plotting unknowns against standard curves for each target.

The reportable range was determined by using seven concentrations of the standard DNA sets as listed in Table 3 ‘ddPCR Copies (Reference)’ and shown in Fig 6. All wells tested in the reportable range generated qPCR signals at every standard level (A, B, C and D). In more highly diluted standards, (Standard D diluted 1:2, 1:4 or 1:8, Standards E, F and G respectively), higher numbers of replicates were run to account for stochastic sampling effects impacted by extremely low copy number input. A signal in at least one well of the replicates was required to be considered within the reportable range of the test. Samples at very low concentration with no signal or Ct were omitted from analysis, as they were presumed to have zero input copies in the well. Some of these samples were later analyzed within the context of the Poisson statistic to show single copy sensitivity (see Fig 5A and 5B and Table 3).

### Master curves–establishing regression formulae and linearity

Target Selector assay linearity and regression formulae were determined using a serial dilution from ddPCR quantified controls that ranged per reaction from > 5000 genomic DNA copies down to a single copy. Testing was executed across 10 independent runs using at least 3 different operators and 5 instruments to obtain a robust performance profile of the test. The master curve regression formulae were developed using four standards (Std A, Std B, Std C, Std D) across 10 plates and grouped by instrument (2 plates per instrument each plate containing 1 curve in triplicate). Samples were included after passing Ct and melting curve criteria. Plotted against the actual (for Stds A-D) or calculated (for standards E-G) ddPCR DNA copy number, the R^2^ value of each of the curves from Fig 6 are listed in S3 Table.

### Validation results summary

In total, we tested 3086 samples for *EGFR*, *BRAF* and *KRAS* Target Selector ctDNA assays for analytical validation, with the *EGFR* WT assay as the background reference. The inter-assay and intra-assay analyses showed r^2^ >0.94–0.99, suggesting a consistent performance among operational variables. Each of Biocept’s Target Selector ctDNA assays showed >99% analytical sensitivity and >99% analytical specificity at a MAF of 0.02% or better. Samples tested from 20 healthy donors (100 tests in total) showed clinical specificity >99%.

### Clinical experience

The analytical validation of Target Selector *EGFR*, *BRAF*, *and KRAS* ctDNA mutation assays and demonstration of detection of single-copy mutant ctDNA are the focus of this work. These assays are laboratory developed tests (LDTs) currently used in a CLIA-certified, CAP-accredited laboratory to evaluate blood from patients with cancer. Clinical validation with extensive sample numbers has yet to be completed where orthogonal evaluation of the patient blood specimens and/or concordance to tissue close to or at the time of blood draw demonstrates testing accuracy in the clinical blood samples. Nevertheless, collective results from CLIA lab testing performed on over 2000 patient samples using the described Target Selector ctDNA assays in the CLIA lab have similar detection rates compared to the prevalence of the corresponding mutations found in the literature. It is important to state that while these clinical experience observations do not constitute a complete clinical validation, the overall data encouragingly reflect comparable mutation detection rates between Target Selector plasma testing and tissue.

Table 8 shows the mutation detection rates using the Target Selector tests in a CLIA-certified, CAP-accredited laboratory setting and are consistent with published mutation incidences. For example, in the case of lung cancer, the prevalence of *KRAS* mutations in tissue ranges from 20–25% in the Western Hemisphere [24]. The Target Selector *KRAS* detection rate in clinical samples is consistent with these values at 20.16%. For *BRAF* V600E, the frequency is 1–2% [25, 26] and the Target Selector *BRAF* detection rate is observed at 1.69%. In a systematic review of 151 studies, the overall frequency of somatic *EGFR* mutations in NSCLC patients with adenocarcinoma histology is approximately 22% in North America (Range: 3%-42%) [27]. In a sampling of over 2000 individuals, the Target Selector ctDNA detection rates for L858R and Del 19 are highly correlative at a prevalence of 20.43%. The Target Selector rate represents 92.87% (20.43%/22%) of the mean frequency for North America (22%) and is especially noteworthy since these two mutations make up approximately 90% of all sensitizing EGFR mutations [28, 29] with a few additional percent comprised of rare mutations (e.g., L861Q, G719A, etc.). This demonstrates a pickup rate for Target Selector that falls within 3% of the mean tissue detection rate at these loci. For NSCLC patients identified with one of these *EGFR* activating mutations, clinical response rates of approximately 75% have been observed with the administration of the EGFR tyrosine kinase inhibitors (TKIs) gefitinib and erlotinib [29]. However, resistance to these TKIs emerge after 6–18 months, where approximately 60% of patients have acquired resistance conferred by the secondary *EGFR* mutation T790M in patients treated with these first and second generation TKIs [30, 31]. The recurrence of the original activating *EGFR* mutation is typically also observed with the emergence of T790M. While treatment histories were unavailable for most of these patients, the observed Target Selector *EGFR* T790M detection rate is 11.81% in Table 8, this value is approximately 58% of the combined detection rates of the L858R and Del19 *EGFR* (20.43%).

**Table 8:** Overall clinical experience of the Target Selector ctDNA platform. Pickup rates for all five targets in a lung cancer population. Columns from left to right show the ‘Target’, total patients tested for each indication ‘Tested’, total number of those patients harboring the target ‘Detected’, and the percent Detected/Tested ‘%MT’ or positive rate.

Lung Testing Summary			
Target	Tested	Detected	% MT
**T790M**	2261	267	11.81%
**L858R**	2261	172	7.61%
**DEL19**	2261	311	13.75%
**BRAF**	1417	24	1.69%
**KRAS**	491	99	20.16%
Del19 + L858R*	2261	462	20.43%

* Cases with at least one activating mutation, Del19 or L858R

In an internal review of collective Target Selector results of patients with lung cancer, slightly over 20% had *KRAS* exon 2 mutations and more than 20% showed activating mutations, whereas only 1.69% of patients had *BRAF* mutations. While relevant tissue status is unavailable for most of these patients, these numbers generally align with the tissue prevalence of these mutations cited in published literature [24, 26, 32–35]. These detection rates can also be influenced by the patient population being tested. For example, treatment status and treatment response can greatly influence the detection of these mutations. Patient ethnicity can also impact pickup rates since patient populations of Asian or Latin descent have been shown to have much higher rates of activating mutations.

Although liquid biopsy technologies are evolving towards cancer screening and early cancer detection, current applications focus on the assessment of patients with advanced cancer. These applications include the following: determining initial biomarker status, monitoring therapeutic response, predicting progression, and providing a complement or alternative to tissue testing when patients are unable or unwilling to undergo invasive methods to obtain tissue.

Further analysis of our clinical results also revealed insights with regards to a cohort of samples derived from Latin America. Specifically, Target Selector *EGFR* ctDNA mutation detection rates in NSCLC from Latin American countries are similar to tissue frequencies presented by Arrieta *et al*. [36]. For instance, in the Arrieta *et al*. paper, tissue prevalence of *EGFR* mutations is cited as 36.7% (95% CI 34.4–39.6) in Mexico from 1417 tested tissue biopsies and 25.2% (95% CI 23.2–27.2) from 1939 tested patients in Columbia. Samples tested in a CLIA-certified, CAP-accredited lab using Target Selector over a 12-month period showed 39.87% positive for *EGFR* activating mutations in Mexico and 26.56% in Columbia, highly correlative with the tissue prevalence from these populations (Table 9). Similar to the test population in Table 8, treatment status in this patient cohort was undefined but the *EGFR* T790M pickup rate, 20.25% for Mexico and 11.72% for Columbia, is close to half the *EGFR* activating mutation rate for each country (39.87% and 26.56% for Mexico and Columbia, respectively). Progressive disease is the primary population for which liquid biopsy is utilized since follow on biopsies are generally difficult to obtain during progression and in these countries. Therefore the observed T790M prevalence is consistent with a population enriched for individuals with lung cancer and progressing on treatment with first or second generation TKIs.

**Table 9:** Clinical experience: EGFR activating mutation frequency from Mexico and Columbia. Clinical Experience Data showing the EGFR activating mutation frequency from Mexican and Columbian patient cohorts at Biocept vs. published tissue prevalence in Latin American countries [36].

**Mexico**				
		**N**	**Positive**	**Percent (95% CI)**
**Biocept Target Selector ctDNA Liquid Biopsy**	T790M	316	64	20.25%
	L858R	316	34	10.76%
	Del19	316	92	29.11%
	Del19 + L858R*	316	126	**39.87%**
				
**Arrieta, *et al*. [36]—Tissue**	Del19 + L858R*	1417	472	**36.70%**
**Columbia**				
		**N**	**Positive**	**Percent (95% CI)**
**Biocept Target Selector ctDNA Liquid Biopsy**	T790M	128	15	11.72%
	L858R	128	12	9.38%
	Del19	128	22	17.19%
	Del19 + L858R*	128	34	**26.56%**
				
**Arrieta *et al*. [36]—Tissue**	Del19 + L858R*	1939	456	**25.20%**

* Cases with at least one activating mutation, Del19 or L858R

## Discussion

Liquid biopsy enables the characterization of cancer biomarkers via the collection of biofluids and provides a systemic representation of a patient’s tumors. Accurate and sensitive methodologies are imperative for ctDNA testing where mutation targets are present at very low copy numbers. Here we describe the analytical validation of ultra-sensitive Target Selector ctDNA assays, sensitive and specific down to a single copy of mutant DNA in a high background of WT DNA. The ability to enrich mutant DNA targets and the extent to which WT DNA amplification is suppressed by Switch-Blocker methodology provides a means for developing exquisitely sensitive assays to identify actionable gene mutations for clinical use. Today, various technologies are used to perform ctDNA liquid biopsy testing, some of which are very sensitive and focused such as ddPCR [37], while others are broad and less sensitive such as Next Generation Sequencing (NGS) with molecular barcodes [38, 39]. Each method has its utility, strengths and weaknesses. While ddPCR is an extremely sensitive methodology it necessitates design and testing for each specific mutation within a hot spot region. For instance, evaluating the most common seven mutations in *KRAS* exon 2 (codons 12 and 13), requires running at least seven separate assays. Input DNA is required for each SNV, which may exhaust the available sample and this does not account for 20 or more rare mutations in KRAS exon 2. In contrast, a single Target Selector assay covers the same two-codon hotspot with an enhanced sensitivity for all SNVs. Similarly, numerous *EGFR* exon 19 deletion mutations can be evaluated using one Target Selector construct and a single reaction, conserving time and valuable patient sample. Target Selector Switch-Blocker technology is versatile and in addition to point mutations, can be adapted to small insertion and deletions, gene fusions and even epigenetic changes when combined with bisulfite sequencing, opening up the possibility for use in early cancer detection and screening applications.

On the other end of the spectrum are the broad based NGS panels. These panels are effective for identifying disease associated drivers and can be valuable in qualifying patients for clinical trials where it is important to identify as many signaling pathways as possible and which could potentially affect disease progression. However, running these large panels can be cost prohibitive and only a few genomic biomarkers on the panel are clinically actionable for patient stratification of first line FDA approved therapies. In the context of standard of care and health economics, these costs may not be warranted for most patients’ needs. Therefore, employing economical testing, like Target Selector assays, that are highly sensitive and focused on actionable biomarkers associated with clinical guidelines can provide a viable and cost-effective strategy to guide treatment decisions and monitor therapeutic response. Inevitably, practical testing for clinical use will employ a balance of speed, coverage, cost and sensitivity. Each of these is successfully addressed by the Target Selector assay which provide a commercial turn-around time of 3–4 days, has good coverage for the most common actionable mutations, is highly economical compared to large NGS panels, can monitor changing mutational profiles over time, and is validated to an ultra-sensitive single copy level.

Various research and commercial ctDNA assays employ different technologies and assay design strategies that directly influences liquid biopsy test sensitivity and costs. For example, while large NGS panels can query multiple gene targets simultaneously, this methodology often provides less sensitive results for individual gene targets and is far more costly to run per reaction than single gene assays. For example, in a recent study by Stetson *et al*. that evaluated four different liquid biopsy NGS panels, a high level of discordance was discovered at variant allele frequencies below 1%. They concluded that this discordance was primarily the result of technical variations in the test methods rather than biological factors [40]. This highlights the need for an extensive validation for each hotspot, something that is difficult if not impossible for large gene panels to achieve. If the treatment decision does not reflect the patient’s mutational profile, it presents a heightened risk to the patient wherein selection of an inappropriate therapy could have potentially serious health consequences.

Health economics are also an important practical consideration in diagnostics. Many community clinics are not realistically able to adopt expensive NGS testing for routine use in prescribing FDA-approved front-line therapies. These first line settings are where the sensitivity, speed, and economic factors of single hot spot assays provide practical advantages and ultimately, patient benefit. NGS excels where multi-gene analysis is needed to examine co-existing pathways driving a patient’s cancer, especially where alternative treatment and/or qualifying a patient for clinical trials is involved. In the United States, a large NGS panel can easily cost thousands of dollars (under Medicare reimbursement pricing) and the list prices can be multiples of that for private insurers. In contrast, Target Selector assay prices range in the few hundreds of dollars (whether Medicare or list price). The intent of precision medicine is to gain actionable information relevant to identifying potential treatment options quickly to tailor the management of a patient’s disease. To this end, accurate and sensitive methodologies are imperative. The assays described herein enable the interrogation of samples with ultra-low mutant allele fractions and are applicable for the detection and monitoring of clinically actionable ctDNA biomarkers relevant for better treatment decisions and patient benefit.

## Materials and methods

### Healthy donor blood collection

Two 8 mL tubes of whole blood were collected from consented healthy donors who were not known to have cancer. Commercially procured whole blood was sourced from Dx Biosamples (San Diego, CA, USA). Blood collection at Biocept with written informed consent was conducted under a study protocol approved by the Western Institutional Review Board (WIRB). Blood was collected in CEE-Sure^TM^ Blood Collection Tubes (BCTs) (Biocept Inc. CA, USA), which contain a patented formula designed to preserve blood cells and ctDNA. Compared to commonly used blood collection tubes such as those containing EDTA or citrate, CEE-Sure^TM^ Blood Collection tubes stabilize nucleated cells and prevent the release of genomic DNA, thus minimizing background WT DNA during ctDNA isolation and follow-on applications. Blood specimens collected in CEE-Sure^TM^ tubes were maintained at room temperature until processing. Plasma was collected after centrifugation at 3,000 x g for 5 minutes at room temperature. A second step of centrifugation of collected plasma was performed at 16,000 x g for 10 minutes at 4°C to pellet any residual cellular debris. The plasma was then transferred to a new tube in preparation for ctDNA extraction.

### Mutation and wild-type DNA sources for analytical validation

DNA from cancer cell lines carrying the specific target mutations were used for analytical assessment. Cell line NCI-H1975 from non-small cell adenocarcinoma carries heterozygous *EGFR* T790M and heterozygous *EGFR* L8585R mutations. Cell line NCI-H1650 from adenocarcinoma carries heterozygous *EGFR* deletion 19 mutation. Cell line NCI-H2122 from non-small cell adenocarcinoma carries homozygous *KRAS* G12C mutation. Cell line SK-MEL-28 from malignant melanoma carries homozygous *BRAF* V600E mutation. All cell lines listed above were obtained from the American Type Culture Collection (ATCC).

WT DNA used in analytical validation of this work was from human placenta (Millipore Sigma, Burlington, MA, USA).

### Clinical sample testing

Analytical validation was performed for each of the assays described in this work prior to their use in a CLIA-certified, CAP-accredited setting as molecular diagnostic laboratory developed tests (LDTs) for patient care. Each clinical sample tested was accompanied by a test requisition form filled by the requesting clinic, stating that the tests were medically necessary for patient care/treatment and that all necessary government, third party payor, and patient consents and approvals were obtained to perform Target Selector testing. Healthcare providers submitted blood specimens for cancer patients at all stages of disease and at any point of treatment (*e*.*g*., treatment naïve, during therapy, treatment progression). Meta-analysis of Target Selector ctDNA testing results was performed to evaluate trends for each assay.

### DNA and ctDNA extraction

Model cell lines were propagated in culture medium with 10% fetal bovine serum according to manufacturer’s instructions. Cells were collected and processed to extract DNA using the QIAamp DNA Mini Kit (QIAGEN, Germantown, MD, USA). Cell-free DNA was extracted from 4 ml of plasma samples using the QIAsymphony DSP Circulating DNA Kit on the QIAsymphony SP instrument (QIAGEN) according to the manufacturer’s instructions. Cell-free DNA was eluted into 100 μL buffer. DNA samples were then quantitated with the Qubit dsDNA HS Assay Kit (Thermo Fisher Scientific, Waltham, MA, USA). Typically, 10 μL of DNA sample was used for each Target Selector assay reaction.

### Target Selector assay

The Target Selector qPCR assays were designed to enrich target mutations in a small hot spot region up to 15 nt in length. For example, one *KRAS* exon 2 blocker covers all variants of both G12 and G13 codons. The Target Selector assays incorporate the QuantStudio 5 (QS5) instrument (Thermo Fisher Scientific, Waltham, MA, USA) for real-time PCR to detect mutations. qPCR was run using a 4-step protocol including denaturation, Switch-Blocker hybridization, primer annealing and template extension steps. Once the mutation is enriched and detected in the sample, Sanger or NGS DNA sequencing is applied to confirm the identity of a target mutation. Similar to probe-based or blocker-based PCR, the Target Selector assay utilizes forward and reverse primers plus a specific blocker labelled with a FAM fluorescent dye. The melting temperature (T_m_) and cycling conditions were optimized based on the amplification primers and blocker for each target. A high-fidelity DNA polymerase was employed to reduce spurious mutations during amplification.

### Reference material generation using ddPCR

Droplet Digital PCR was performed primarily on a QX100 from Bio-Rad. Following the isolation of cell line DNA or acquisition of human placental DNA, 10ul of 2x ddPCR supermix for Probes (No dUTP) (Bio-Rad Cat#1863024), 1ul 20xPrimers and Probe of Target Assay* and DNA template plus nuclease free water were added up to 20ul total volume per well. Each reaction was mixed with DG oil (Bio-Rad Cat#1864110) according to manufacturing protocol and run through Droplet Generator then emulsion was transferred to 96 wells plate and performed PCR for 40 cycles (Step1: 95C 10 min, step2: 94C 30 sec then 55C 1min for 40x, step3: 4C indefinitely) on Thermocycler ABI9700. The PCR plate was read in Droplet Reader and copy number of sample was determined. Assays used for copy number determined were from BioRad and included: BRAF (V600E dHsaCP2000027, WT dHsaCP2000028); EGFR E746_A750del dHsaCP2000039, WT dHsaCP2000040), EGFR (L858R dHsaCP2000021, WT dHsaCP2000022); EGFR (T790M dHsaCP2000019, dHsaCP2000020, WT), KRAS (G12C dHsaCP2000007, dHsaCP2500585, WT)

### Analytical specificity assessment

WT DNA extracted from human placenta (Millipore Sigma, Burlington, MA, USA) was used to test the analytical specificity of each Target Selector assay. For each target, we tested 112 reactions where each reaction contained 14,000 copies of WT DNA. Quality control metrics including T_m_ range were implemented to improve the target specificity.

### Analytical sensitivity assessment

The analytical sensitivity of the Target Selector assay was evaluated with cell line DNA carrying specific target mutations. Mutant DNA was tested in the presence or absence of WT DNA. For each target, we tested at least 120 reactions with various defined mutant DNA copy numbers. The same quality control metrics as described above were applied.

DNA standards were created by determining copy numbers by ddPCR using the QX200 instrument (Bio-Rad, Hercules, CA, USA). To determine the limit of detection (LOD), mutant DNA was spiked into 14,000 copies of WT DNA. Mutant DNA was diluted from 8 copies at a two-fold serial dilution down to 1 copy: Standard D = 8 copies, Standard E = 4 copies, Standard F = 2 copies, and Standard G = 1 copy. The detection rate from the serial dilution of very low mutation copy numbers was compared to the Poisson distribution which predicts the theoretical probability of a specific number of events within a fixed space. Following quantitation of each input standard, the statistical method of maximum likelihood estimation (MLE), was independently applied to estimate the mutant copy number at each standard level determined by qPCR results.

### Reportable range, repeatability and reproducibility test

A broad reportable range of mutant DNA copies per reaction was tested from ~32,000 copies down to one copy. Analytical validation was conducted by three independent operators using five QS5 instruments across five days in Biocept’s CLIA-certified and CAP-accredited laboratory. A 4-point standard curve of mutant DNA at 8-fold dilutions was run in triplicates. Prior to use for reportable range, repeatability and reproducibility experiments, all standards were prepared and qualified by ddPCR to verify copy number inputs of the control materials. In addition, triplicate negative control reactions were run for NTC and 14,000 copies of WT human placental DNA. The experiments were completed on non-consecutive days by three different laboratory technologists.

### Clinical specificity

For clinical specificity, 20 healthy donors were tested for each target. Whole blood samples were collected in CEE-Sure^TM^ BCT. DNA extraction from 4 ml of plasma was performed using the QIAsymphony DSP Circulating DNA Kit with the QIAsymphony SP instrument (QIAGEN). A Hamliton Star automated liquid handler (Hamilton Inc. Reno, NV) was used to set up qPCR reactions. The same quality control metrics as in the specificity assessment were applied. In addition to target mutation assays, the *EGFR* WT assay was performed to assess the loading quantity and overall mutation allele frequency.

## Supporting information

S1 Table Inter-assay reproducibility. Inter-assay precision aggregated as means of plate (1–10). Columns are: Assay.Standard–Assay name, Standard (A,B,C, or D), mean of means Ct values aggregated by plate, standard deviation of means of Ct values aggregated by plate, percent Coefficient of Variation. All inter-assay standard deviations fell within the 1 Ct standard deviation set as the acceptance criteria.
(XLSX)

S2 Table Intra-assay reproducibility. Intra-assay reproducibility aggregated as means of each plate (1–10). Columns are: Assay.Std.Inst—Assay name, Standard (A, B, C or D), Instrument, mean of Ct values by plate, standard deviation of Ct values by plate, percent Coefficient of Variation for each set of replicates. 58 of 60 intra-assay standard deviations fell within the 1 Ct standard deviation except for a single set in EGFR WT Std D and a single set in T790M Std D.
(XLSX)

S3 Table Regression curves and linearity. Data used to generate master standard curves and assay performance Data for 10 standard curves run across 5 instruments is shown by instrument number as indicated after the target name. Standard curves features for the y.intercept, slope, efficiency and r squared values are published along with the aggregate values use to form the master standard curve which is highlighted below the instrument level data. S2 Table Intra-Assay Reproducibility.
(XLSX)
